# Distribution of uterocervical angles of pregnant women at 16^+ 0^ to 23^+ 6^ weeks gestation with low risk for preterm birth: first vietnamese cohort of women with singleton pregnancies

**DOI:** 10.1186/s12884-023-05597-3

**Published:** 2023-04-28

**Authors:** Thi Hoang Trang Nguyen, Van Tam Vu, Vu Quoc Huy Nguyen

**Affiliations:** 1 Department of Obstetrics and Gynecology, Haiphong University of Medicine and Pharmacy, 72A Nguyen Binh Khiem St, Haiphong, Vietnam; 2 Department of Obstetrics and Gynecology, Hue University of Medicine and Pharmacy, Hue University, 6 Ngo Quyen St., Hue 491200, Hue, Vietnam

**Keywords:** Uterocervical angle (UCA), Singleton pregnancy, Preterm birth

## Abstract

**Background:**

Cervical length (CL) measured by ultrasound in the second trimester is a predictor of spontaneous preterm birth (sPTB). The uterocervical angle (UCA) has recently been suggested as a predictor to identify women at risk of sPTB. The aim of this study was to investigate the UCAs’ distribution in singleton pregnant women at 16^+ 0^ − 23^+ 6^ weeks of gestation with low risk for sPTB.

**Methods:**

This was a prospective cohort study of 1,051 pregnant women with singleton pregnancies at low risk for preterm delivery. Pregnant women with a viable singleton fetus at 16^+ 0^ − 23^+ 6^ weeks of gestation were enrolled in the study conducted at the Haiphong Hospital of Obstetrics and Gynecology, Vietnam, from 09/2019 to 09/2020. CL and the UCA were assessed using transvaginal ultrasonography (TVS) by a single sonographer. Subjects were followed-up until the end of pregnancy, and maternal and neonatal outcomes were recorded. The UCAs’ range and their relationship with gestational age were evaluated using regression analysis. P < 0.05 was considered statistically significant.

**Results:**

The normal range of the UCA (5^th^ − 95^th^ percentiles) was 46.47° (95% CI, 40.27°-51.81°) to 127.06° (95% CI, 123.02° − 130.71°). The UCAs in the preterm birth (< 37 weeks) and full-term groups were 117.86° ± 20.25° and 83.80° ± 24.18°, respectively (p < 0.001). Linear regression analysis showed a significant change in the UCA range from 16^+ 0^ to 23^+ 6^ weeks of gestation (2.51 degrees per week, p < 0.001). The linear function yielded the highest correlation coefficient in the variation rule of the UCA values (r = 0.22). A total of 42/63 (66.7%) patients with preterm birth < 37 weeks had a UCA above the 75^th^ percentile. The majority of women with preterm birth had a UCA ≥ 95° compared with those with full-term delivery (88.9% vs. 31.3%, p < 0.001).

**Conclusions:**

The results of this study present background information about the normal range of UCA values in singleton pregnant women at 16^+ 0^ to 23^+ 6^ weeks at low risk for sPTB in this Vietnamese cohort. In this study population at low risk for sPTB, pregnant women with a UCA value ≥ 95^o^ were also considered at risk for preterm birth.

**Supplementary Information:**

The online version contains supplementary material available at 10.1186/s12884-023-05597-3.

## Background

According to the World Health Organization, a preterm birth (PTB) is defined as a live birth occurring between 20^+ 0^ and 36^+ 6^ gestational weeks [1]. Globally, the prematurity rate is 10.6%, resulting in nearly one million neonatal deaths each year [2, 3]. In Vietnam in 2014, the data showed a PTB rate of 9%, ranking Vietnam 21st in the world [4]. Preterm birth is a major cause of neonatal morbidity and mortality, mostly due to immature respiratory organs, cerebral hemorrhage and infection, which can lead to long-term neurological deficits such as intellectual impairment, cerebral palsy, chronic lung disease, deafness and blindness [3, 5]. Approximately one-third of all preterm births are medically indicated, and the rest occur spontaneously, which remains a challenge in obstetric care [6]. Early identification of subjects at risk of spontaneous preterm birth (sPTB) from the general pregnant population is essential for offering adequate prevention measures. Many strategies have been developed to predict and prevent spontaneous prematurity. Until now, a previous history of sPTB and a short cervix were the main screening criteria [7, 8]. Sonographic cervical length measurement has been consistently shown to be an efficient and cost-effective strategy in the prediction of sPTB in asymptomatic singleton pregnant women [9–12]. A cervical length (CL) cut-off ≤ 25 mm by transvaginal ultrasound is considered a strong risk predictor of preterm birth in singleton pregnant women. However, its detection rate for spontaneous preterm deliveries at < 34 weeks is only approximately 55%, with a 10% false-positive rate [13, 14]. Therefore, additional screening parameters are needed to identify pregnant women at risk of preterm birth to provide timely preventive measures.

The uterocervical angle (UCA) has recently been studied as a parameter to identify women at risk for sPTB [15]. If the UCA is more obtuse, the gravity of the uterus and the fetus acting on the internal os tends to be along the direction of the cervix, which can lead to shortening of the cervix, and this is one of the factors causing preterm birth [16, 17]. UCA measurement, performed by transvaginal ultrasonography (TVS) during the second trimester of gestation, has been reported as a high-performance screening tool in predicting preterm birth [18, 19]. Studies by Dziadosz et al. [19] and Knight et al. [20] found that the combination of UCA with cervical length measurements provides a stronger predictor of preterm birth. A recent study by Luechathananon et al. [21], the first prospective observational cohort study of its kind, showed that in subjects with threatened preterm labor and a mean gestational age of 35^+ 0^ (range, 33^+ 0^, 36^+ 0^) weeks, UCA measurement by using TVS can be considered a useful tool for predicting preterm birth. Moreover, there is still a lack of in-depth studies evaluating the real-life distribution of UCA values in pregnant women with term or preterm deliveries, and there is still no consensus on the appropriate gestational age during the second trimester at which to perform UCA measurement to identify women at risk of preterm birth. This study aimed to investigate the distribution of UCA values in singleton pregnant women at 16^+ 0^ − 23^+ 6^ weeks gestation with low risk for sPTB from a cohort consisting of women with term and preterm deliveries.

## Methods

### Ethical considerations and study design

The research proposal was approved by the Ethical Council in Biomedical Research of Hue University of Medicine and Pharmacy, Vietnam (Ethics Committee ID number H2020/035) and the Scientific Council of Haiphong Hospital of Obstetrics and Gynecology, Vietnam (IEC, 1186/QD-BVPSHP). All participants voluntarily signed a written informed consent form after hearing a full explanation of the purpose of this study.

This study was a longitudinal cohort study conducted from September 2019 to September 2020 at the Department of Pregnancy Management and Prenatal Diagnosis of Haiphong Hospital of Obstetrics and Gynecology, Vietnam.

### Sample size calculation

The sample size of this study was estimated using the following formula:

$${N}=\frac{{{z^2}{{(1 - \frac{\alpha }{2})}^{{S^2}}}}}{{{{(\overline X .\delta )}^2}}}{\times}{L}$$

L: number of gestational age groups; there were 8 groups from 16^+ 0^ to 23^+ 6^ weeks of gestation.

Z_(1-α/2)_ = 1.96, δ value = 0.025, ${\overline X}$: mean of UCA, *S*: standard deviation. According to Singh et al. [22], ${\overline X}$ = 88.4 degrees and *S* = 6.81 degrees. Based on these values, the minimum sample size was 292 subjects.

### Study population

All singleton pregnant women aged 18 to 40 years old and between 16^+ 0^ and 23^+ 6^ weeks of gestation with viable fetuses who were examined and managed at the Department of Pregnancy Management & Prenatal Diagnosis of Haiphong Hospital of Obstetrics and Gynecology between September 2019 and September 2020 were included in the study.

Gestational age was determined from the menstrual history and confirmed by the fetal crown-rump length at the first-trimester ultrasound examination for patients who conceived naturally and by the date of embryo transfer or intrauterine insemination for those who conceived after Assisted Reproductive Technology.

The exclusion criteria were as follows: (1) a history of sPTB or second trimester miscarriage (miscarriage at 13^+ 0^-19^+ 6^ weeks gestation) [23], (2) a short CL (CL ≤ 25 mm), (3) signs of threatened miscarriage or preterm birth, (4) severe fetal malformations, (5) medically indicated preterm birth, (6) a cervical mass or previous cervical surgery, (7) the use of available preterm birth prevention methods (micronized progesterone, cerclage, cervical pessary), and (8) loss of follow-up.

**Fig. 1:**
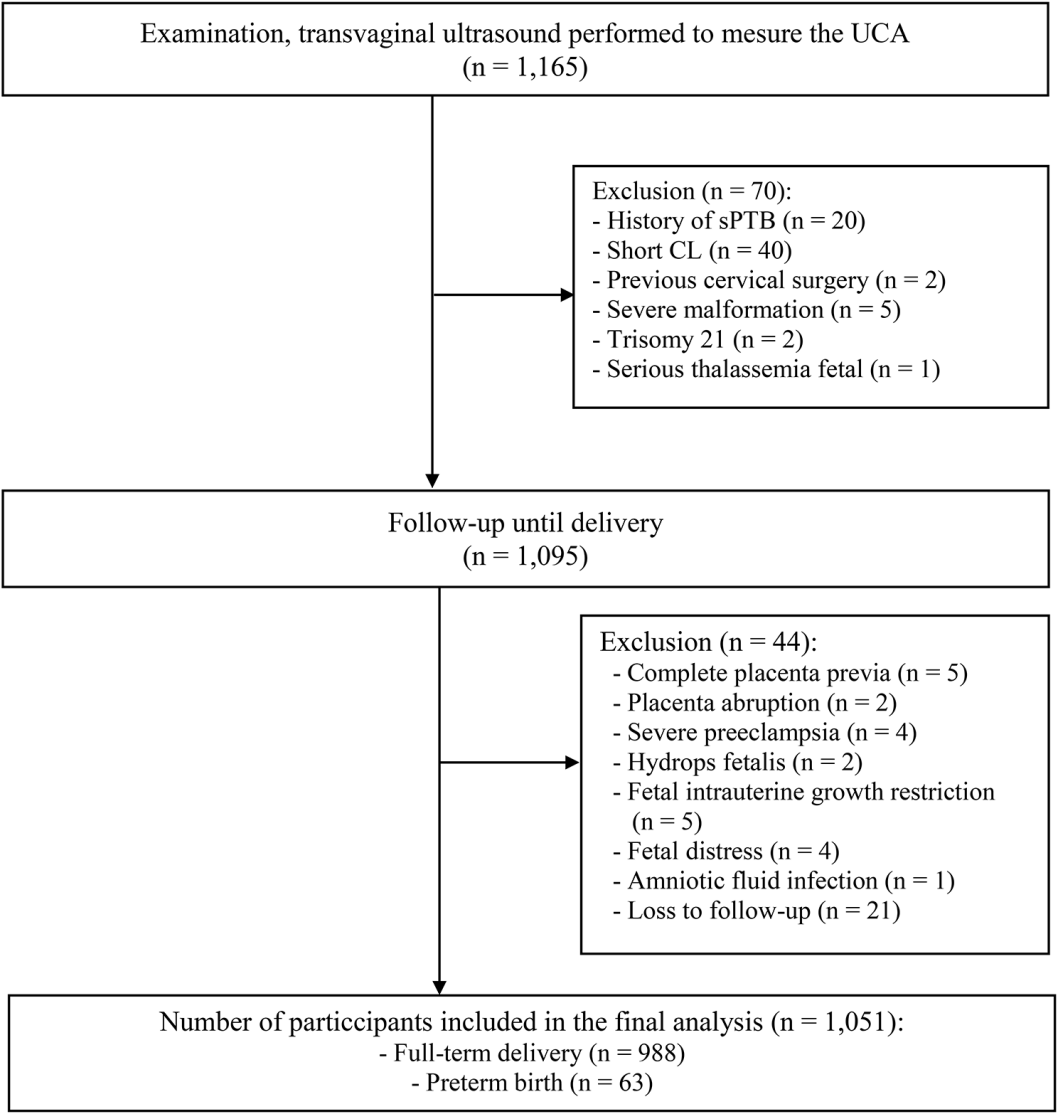
Study diagram

A total of 1,165 pregnant women with singleton pregnancies at 16^+ 0^ to 23^+ 6^ weeks gestation were voluntary participants in this study and were recruited according to the recruitment guidelines. Each participant underwent TVS once for CL and UCA measurements and was followed-up until delivery. Women who delivered at other hospitals were contacted via telephone. After excluding 114 participants at high risk for sPTB or loss of follow-up, 1,051 pregnant women were included in the final analysis (Fig. 1).

### Assessment of cervical length and the uterocervical angle

**Fig. 2:**
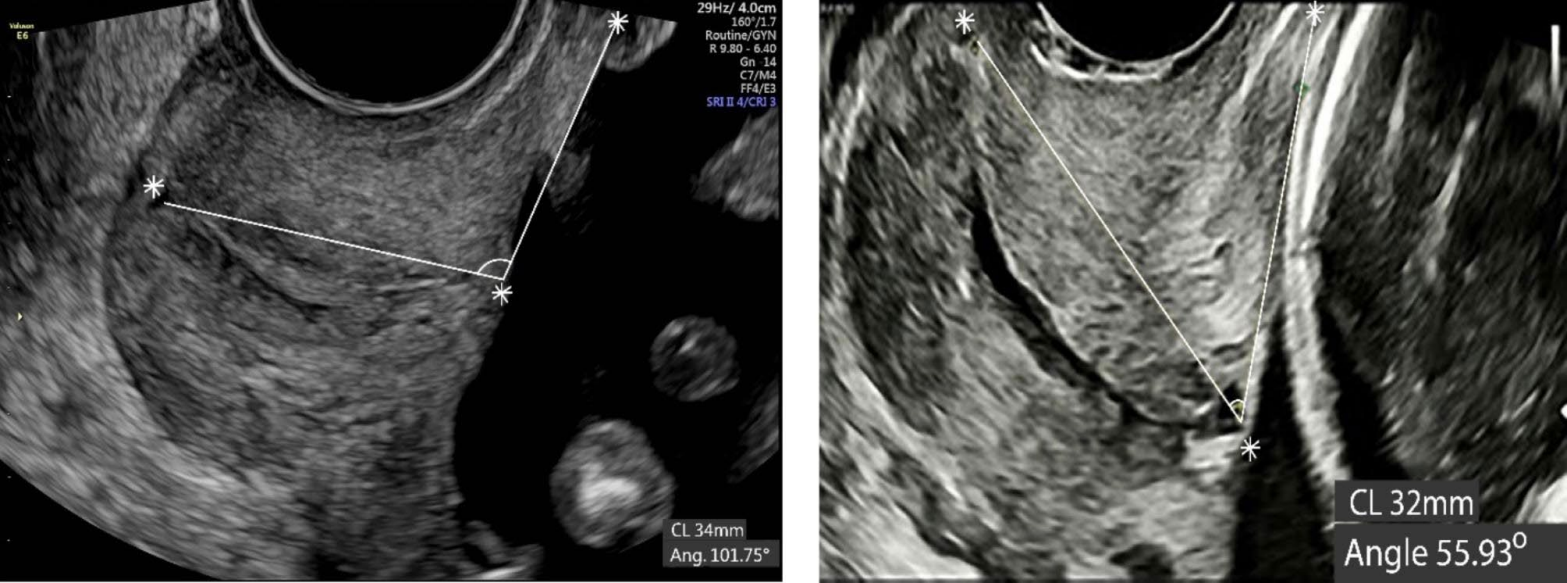
(a) GA 20^+ 0^ weeks, UCA 101.75^o^. (b) GA 22^+ 2^ weeks, UCA 55.93^o^. Transvaginal ultrasound measurement of the UCA. Measurement of the triangular segment between the lower uterine segment and the cervical canal

The cervical length and uterocervical angle measurements were performed by a single sonographer who was certified and monitored by the Maternal Fetal Medicine Foundation. The ultrasound machines used for measurements were the Samsung Medison WS80A (Korea) and GE Voluson E6 (GE Healthcare Korea) with a transvaginal probe (frequency 4.0–9.0 MHz). Patients had an empty bladder, and excessive pressure on the cervix was avoided. The CL measurements were performed following the standard method of The Fetal Medicine Foundation, tracing a single straight line from the internal to the external os [24]. The UCA was measured following previously published protocols, according to the method described by Dziadosz et al. [19]. In short, a first line is placed from the internal os to the external os irrespective of whether the cervix is straight or curved. A second line is then drawn to delineate the lower uterine segment. Ideally, the second line reaches 3 cm up the lower uterine segment to establish an adequate measurement. The angle between the two lines is the UCA value. Specifically, in the study, we measured the UCA along with the CL, on the same cross-section image. We first drew the cervical line and measured CL, which was at least 25 mm in our study population. Then, we measured the UCA, of which the second side length was estimated at least 3 cm according to the CL (Fig. 2).

Each participant had three images measured to reduce measurement bias, and the most obtuse UCA from the three images was used. The patients’ demographic characteristics, ultrasound images, expected date of delivery, obstetric complications and perinatal outcome data were recorded.

### Outcome measures

The percentile chart of the singleton pregnant woman at 16^+ 0^ to 23^+ 6^ weeks gestation with low risk for sPTB was defined as the primary outcome parameter. The secondary outcome was the percentage of preterm birth women before 37 weeks that had UCA values ≥ 95^o^ and lying on the 75^th^ percentile curve. Pregnant women with a UCA value ≥ 95^o^ were considered at risk for preterm birth, according to the previous studies [19, 22].

### Statistical analysis

All analyses were performed using SPSS version 26.0 (SPSS, Inc., Chicago, IL).

Student’s t test was used to evaluate the difference between two means, and p < 0.05 was considered statistically significant. Calculating the correlation between two quantities according to each function y = f(x) (y is anthropometric quantities, x is gestational age), there was a correlation when r > 0.5. The distribution of UCA values was visualized using a scatter plot against gestational age. Predicted median and 5^th^ and 95^th^ percentiles of UCA values with 95% confidence intervals according to GA were estimated using quantile regression, which allows the possibility to detect whether the range of UCA values changes with GA, as well as the display of the confidence band around each percentile. Kurtosis and skewness calculations were performed to determine the distribution of cervical angle measurements according to gestational age which is normal when the Kurtosis coefficient ≤ ± 2 and skewness coefficient ≤ ± 2. These distribution characteristics were determined to calculate the values corresponding to the percentile curve. If the distribution was normal, the percentile curve was measured by the following formula: percentile curve = x ± k.SD [25, 26]. The mean values were determined after solving the selective equation (with the highest r), and the values corresponding to the percentiles calculated according to the above formula were the basis for establishing the UCA percentile chart according to gestational age.

## Results

A total of 1,165 pregnant women were included in this cohort. During the follow-up period, 114 women at high risk for sPTB or loss to follow-up were identified and excluded from the final analysis. The exclusion criteria were as follows: short cervical length (40 women), history of sPTB (20 women), complete placenta previa (5 women), placental abruption (2 women), severe preeclampsia (4 women), hydrops fetalis (2 women), severe fetal malformations (5 women), fetal chromosomal abnormality (2 with trisomy 21), fetal intrauterine growth restriction (5 women), serious thalassemia fetal (1 woman), fetal distress (4 women), amniotic fluid infection (1 woman), previous cervical surgery (2 women), and loss to follow-up (21 women). Overall, 1,051 pregnant women who met the study criteria were divided into two groups: a full-term delivery group (≥ 37 weeks, 988 women) and a preterm delivery group (before 37 weeks, 63 women). A full comparison of the demographic and clinical data of the two groups is presented in Table 1. The mean UCA value increased with GA from 16^+ 0^ to 23^+ 6^ weeks (Table 2), and the difference was statistically significant (p < 0.001).

**Table 1:** Study subjects’ characteristics

Characteristics	Full-term delivery (≥ 37 weeks) (n = 988)	Preterm birth < 37 weeks (n = 63)	P value*
***Maternal characteristics***			
Age (years)	28.83 ± 5.06	29.19 ± 5.02	0.5913
BMI (kg/m^2^)	20.53 ± 2.51	20.37 ± 2.07	0.676
Parity	1.2 ± 0.5	1.3 ± 0.7	0.2685
Gestational age at TVS (weeks)	19.74 ± 2.31	20.90 ± 1.79	< 0.001
CL (mm)	36.52 ± 5.07	32.05 ± 4.37	< 0.001
UCA (degrees)	83.80 ± 24.18	117.86 ± 20.25	< 0.001
UCA ≥ 95^o^	309 (31.3%)	56 (88.9%)	< 0.001
***Neonatal characteristics and outcomes***			
Gestational age at birth (weeks)	38.41 ± 0.90	34.36 ± 2.01	< 0.001
Birthweight (gram)	3182.79 ± 285.42	2412.69 ± 480.42	< 0.001
C-section	410 (41.5%)	8 (12.7)	< 0.001
NICU admission	34 (3.44)	33(54.10)	< 0.001
Deaths	0	2	NA

*P-values were obtained by Chi-square test for categorical variables and T-test for continuous variables. SD: standard deviation; BMI, body mass index; TVS: Transvaginal ultrasound; UCA: uterocervical angle; NICU: Neonatal Intensive Care Unit; NA: not applicable.

**Table 2:** Mean value of the UCA at 16^+ 0^ to 23^+ 6^ weeks gestation

GA	N	Mean	SD
16^+ 0^ − 16^+ 6^	112	74.65	22.19
17^+ 0^ − 17^+ 6^	110	79.15	24.76
18^+ 0^ − 18^+ 6^	149	81.07	27.32
19^+ 0^ − 19^+ 6^	82	88.30	20.05
20^+ 0^ − 20^+ 6^	110	86.96	24.15
21^+ 0^ − 21^+ 6^	145	90.71	23.22
22^+ 0^ − 22^+ 6^	215	89.82	26.93
23^+ 0^ − 23^+ 6^	128	92.21	24.32

p < 0.001

In the preterm birth group, the mean CL was significantly shorter (36.52 ± 5.07 mm vs. 32.05 ± 4.37 mm, p < 0.001), and the mean UCA value was significantly wider than those in the full-term group (83.80 ± 24.18° vs. 117.86 ± 20.25°) (p < 0.001). There was no significant difference between the two groups in regard to maternal age, BMI, and the parity (p > 0.05).

The results of the Kurtosis coefficient and skewness coefficient of the UCA value according to GA subgroups are described in Table 3.

**Table 3:** Kurtosis coefficient and skewness coefficient of the UCA at 16^+ 0^ to 23^+ 6^ weeks gestation

GA	Kurtosis coefficient	Skewness coefficient
16^+ 0^ − 16^+ 6^	0.247	0.281
17^+ 0^ − 17^+ 6^	0.550	1.193
18^+ 0^ − 18^+ 6^	0.092	0.318
19^+ 0^ − 19^+ 6^	0.430	0.783
20^+ 0^ − 20^+ 6^	0.345	0.168
21^+ 0^ − 21^+ 6^	0.119	-0.149
22^+ 0^ − 22^+ 6^	0.322	1.384
23^+ 0^ − 23^+ 6^	0.071	0.095

**Fig. 3:**
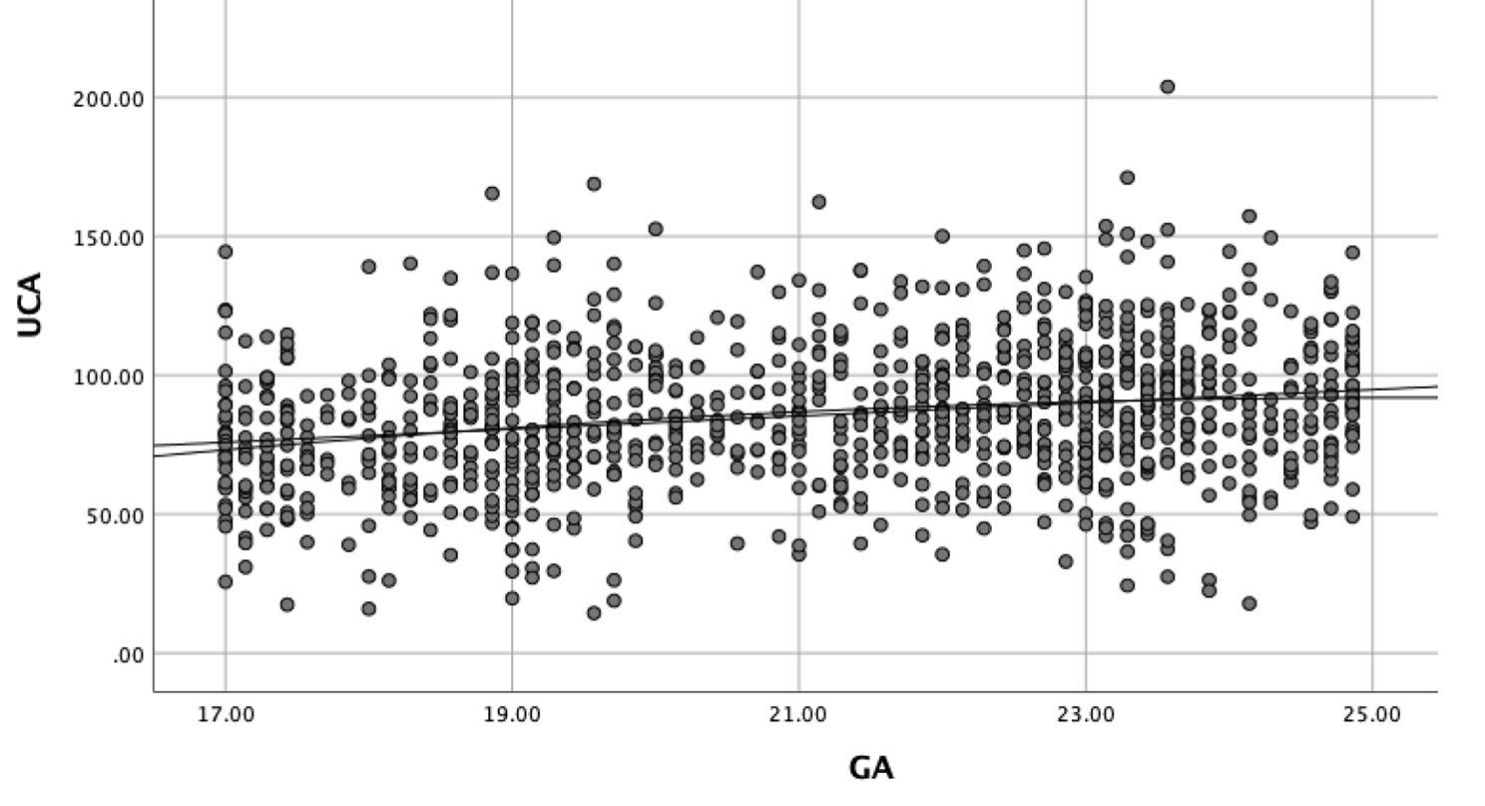
Distribution of UCA values according to gestational age

To demonstrate and determine the rule of the UCA measurement variation with a GA from 16^+ 0^ to 23^+ 6^ weeks, we determined the relationship between the UCA value (y) and GA (x) according to a linear function, a quadratic function and a cubic function. The function with the highest correlation coefficient correctly represented the variation rule of UCA values, which was the linear function (r = 0.22). The line representing the UCA variation rule connects the mean values after solving the linear function, y = 35.58 + 2.37x (Fig. 3).

Based on the normal distribution, Table 4 presents the mean values and corresponding values for the 5th, 10th, 25th, 50th, 75th, 90th, and 95th percentile curves.

**Table 4:** GA-based UCAs corresponding to the 5th, 10th, 25th, 50th, 75th, 90th, and 95th

GA	N	SD	Distribution of UCA values according to percentile (^o^)						
			5%	10%	25%	50%	75%	90%	95%
16^+ 0^− 16^+ 6^	112	22.19	39.82	48.11	59.32	73.55	88.62	104.64	114.10
17^+ 0^− 17^+ 6^	110	24.76	45.13	50.73	61.14	79.59	91.79	105.94	127.75
18^+ 0^− 18^+ 6^	149	27.32	30.03	45.32	64.80	79.86	100.39	114.50	124.44
19^+ 0^− 19^+ 6^	82	20.05	58.23	66.28	73.17	85.60	101.31	113.52	125.20
20^+ 0^− 20^+ 6^	110	24.15	48.72	56.00	70.90	85.68	102.07	119.73	132.69
21^+ 0^− 21^+ 6^	145	23.22	52.53	59.58	74.90	87.93	106.28	119.33	131.28
22^+ 0^− 22^+ 6^	215	26.93	44.30	57.84	72.44	90.37	105.14	122.35	128.48
23^+ 0^− 23^+ 6^	128	24.32	53.00	60.84	75.24	91.20	109.58	122.83	132.56

Linear regression analysis showed a significant change in the range of UCA values from 16^+ 0^ to 23^+ 6^ weeks of gestation (increase of 2.51 degrees per week, p < 0.001, Fig. 3). The range of the UCA at the 5th to 95th percentile ranges from 38.96° (95% CI, 35.45° − 44.31°) to 133.70° (95% CI, 128.92° − 139.32°).

**Fig. 4:**
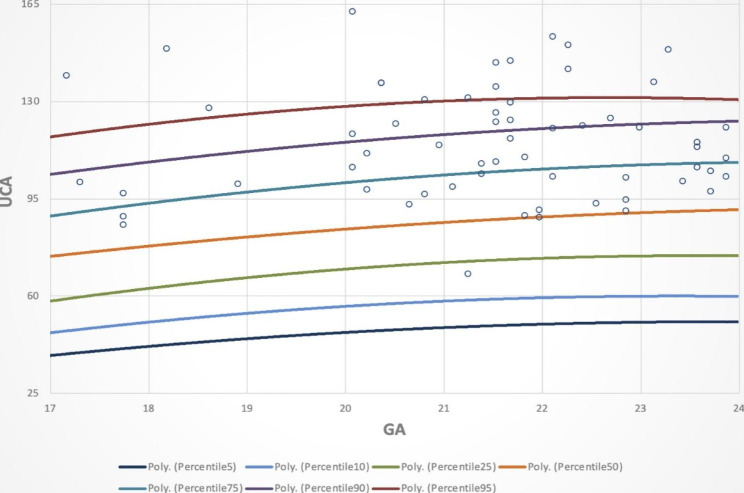
Distribution of UCA values in the preterm birth <37 weeks group

There were 42/63 women with preterm birth before 37 weeks with a UCA value above the 75th percentile and 56/63 women with preterm birth before 37 weeks with a UCA value ≥ 95°, accounting for 66.7% and 88.9%, respectively (Fig. 4).

## Discussion

Our study on 1,051 singleton pregnant women at 16^+ 0^ to 23^+ 6^ weeks gestation with low risk for sPTB who were treated at Haiphong Hospital of Obstetrics and Gynecology from September 2019 to September 2020 showed that the normal range of UCA values at the 5th to 95th percentile was from 46.47° (95% CI, 40.27° − 51.81°) to 127.06° (95% CI, 123.02° − 130.71°) (Fig. 4), with significant changes during this GA period (increase of 2.51 degrees per week, p < 0.001, Fig. 3). Similarly, the study of Llobet et al. [27] with 275 singleton pregnant women showed that the mean UCA values increased from the first to the second trimester with statistical significance (84.2° versus 94.5°, p = 0.019). Sawaddisan et al. [28] (Thailand, 2020) studied 372 singleton pregnant women at GAs from 16^+ 0^ to 23^+ 6^ weeks and showed that UCA values changed according to GA, but this change was not statistically significant (increase of 0.3° per week, p = 0.757).

As shown in Table 1, the mean values of the UCA in the preterm birth before 37 weeks group were significantly wider than those in the full-term delivery group (83.80° ± 24.18° vs. 117.86° ± 20.25°, p < 0.001). Sochacki-Wojcicka et al. [29] also found that there was a statistically significant difference in the mean UCA value between the preterm birth group and the full-term delivery group in the first trimester (115.5° vs. 85°, p = 0.0002) and 2nd trimester (126° vs. 91.5°, p < 0.0001). The study of Llobet et al. [27] with the aim of determining the correlation of the cervical angle with preterm birth also concluded that the UCA in the second trimester in the preterm birth group was wider than that in the full-term delivery group [(105.16° vs. 94.53°, p = 0.015, RR = 0.821 (95% CI, 0.74–0.97)]. Table 1 also demonstrates that the rate of preterm birth before 37 weeks in our study was 6% (63/1051 women). The majority of women with preterm birth had a UCA ≥ 95° compared with those with full-term delivery (88.9% vs. 31.3%, p < 0.001).

Recently, several studies have shown that the UCA can be useful in predicting sPTB [19, 22, 29–31]. Dziadosz et al. [19] demonstrated in their study that UCA performed better than CL in predicting sPTB with higher sensitivity and negative predictive value (NPV). A UCA ≥ 95° was significantly associated with sPTB < 37 weeks with a sensitivity of 80% (p < 0.001, CI 0.70–0.81, NPV 95%). A UCA ≥ 105° predicted sPTB < 34 weeks with a sensitivity of 81% (p < 0.001, CI 0.72–0.86, NPV 99%). CL ≤ 25 mm significantly predicted sPTB < 37 weeks (p < 0.001, sensitivity 62%, NPV 95%) and < 34 weeks (p < 0.001, sensitivity 63%, NPV 97%). The authors concluded that the combination of both CL and UCA measurements may be the best predictor of risk of sPTB. A recent study by Singh et al. [22] also revealed that the risk of spontaneous preterm delivery was higher in women with obtuse UCAs (≥ 95 degrees), with a sensitivity of 86.7%, specificity of 93.0%, positive predictive value of 83.0%, and negative predictive value of 94.6%. It can be explained that if the UCA is obtuse, the gravity of the uterus and the fetus acting on the internal os tend to be along the direction of the cervix, which can lead to shortening of the cervix, and this is one of the factors causing preterm birth. Therefore, the function of cervical cerclage based on this mechanism is not only to support the cervix to evenly distribute the force from the uterus but also to change the UCA from obtuse to acute, changing the force of the uterus to the posterior fornix to avoid shortening the cervix. This has been proven through the study of Cannie et al. [16]. They analyzed CL and UCA measurements in 2 groups of pregnant women: 198 women with no high risk of preterm birth and 73 women with a high risk of preterm birth who had an Arabin pessary at 14 to 33 weeks gestation. The authors performed cervical magnetic resonance imaging before inserting the Arabin pessary and monthly follow-up until the pessary was removed. The results showed that in the group of pregnant women with a low risk of PTB, the UCA measurement did not change, but the CL values decreased significantly with GA (r = − 0.15, p < 0.05). In the high-risk preterm birth group, 63 patients who delivered after 34 weeks had a mean UCA value that was significantly reduced compared to that before the Arabin pessary was inserted (132° vs. 146°, p < 0.01), but it did not change in the 8 patients who delivered before 34 weeks (143° vs. 152°, p > 0.05).

To demonstrate the clinical applicability of the established UCA percentile chart, we performed the distribution of UCA in the PTB before 37 weeks gestation group on the percentile chart of the singleton pregnant woman at 16^+ 0^ to 23^+ 6^ weeks gestation (Fig. 4), and we found that most of the women with preterm birth before 37 weeks had a UCA value above the 75th percentile (42/63 women, accounting for 66.7%). Based on the results of this study, we have the same opinion as some authors that the UCA values in women with PTB is wider than that in women with term delivery. Preterm birth rates are increased in women with obtuse uterocervical angles. Thus, should we consider the 75th percentile on the above UCA percentile chart as a limit to predict PTB before 37 weeks in pregnant women at low risk for preterm delivery? More in-depth studies with a large sample size are necessary to prove the prognostic value of UCA measurements in the prediction of preterm birth, especially in combination with a short cervical length.

This study had three major strengths. First, this is the first study in Vietnam to establish the percentile chart of UCA measurements in singleton pregnant women at 16^+ 0^ to 23^+ 6^ weeks gestation with low risk for sPTB. Second, the measurement of all uterocervical angles was performed by a single obstetrician to control for interobserver variability, and the prospective nature of the study to control for the risk of information bias, focusing on investigating the UCA values of a large study sample, can also be considered strengths of the study. Third, the study subjects included only pregnant women at low risk for sPTB (without a history of sPTB or short cervical length), which could have eliminated the role and impact of these factors on pregnancy outcomes. However, the present study had several limitations. First, women with several maternal conditions that predispose women to sPTB, such as a history of sPTB, short cervical length, and previous cervical surgery, were excluded from the study sample, limiting the representativeness of the general population of pregnant women. Second, selecting pregnant women from a single center can affect the generalizability of our findings. Third, we have not yet in this study assessed the intra-observer variability of UCA’s measurement.

## Conclusions

The results of this study present background information about the normal range of UCA values in singleton pregnant women at 16^+ 0^ to 23^+ 6^ weeks at low risk for sPTB in this Vietnamese cohort. In this study population at low risk for sPTB, pregnant women with a UCA value ≥ 95^o^ were also considered at risk for preterm birth.

## Electronic supplementary material

Below is the link to the electronic supplementary material.

Additional file 1

Additional file 2

## Data Availability

The dataset used and/or analyzed during the current study can be found in the Additional files.

## List of Abbreviations

CL: Cervical length
UCA: Uterocervical angle
TVS: Transvaginal ultrasonography
PTB: Preterm birth
sPTB: Spontaneous preterm birth
GA: Gestational age
