# Supplementary material for: Distribution of uterocervical angles of pregnant women at 16+ 0 to 23+ 6 weeks gestation with low risk for preterm birth: first vietnamese cohort of women with singleton pregnancies
Source: BMC Pregnancy Childbirth. 2023 Apr 28;23:301. doi: 10.1186/s12884-023-05597-3 (PMC10148387; doi:10.1186/s12884-023-05597-3)
Supplement: Supplementary file 2 — Additional file 2 [file 12884_2023_5597_MOESM2_ESM.docx]

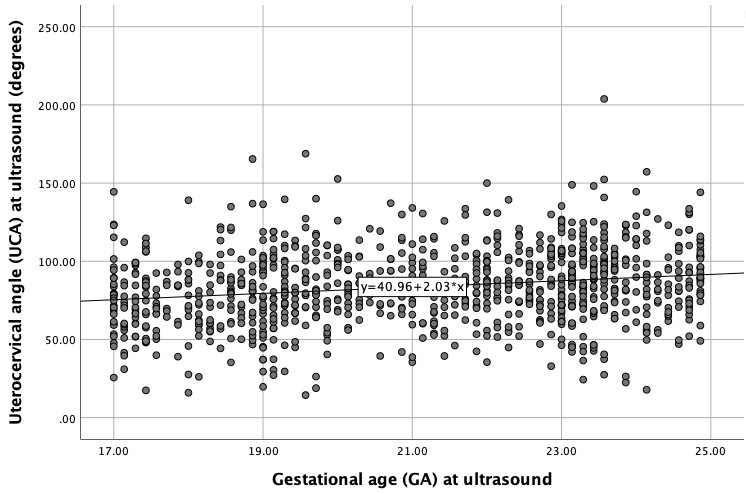


**Figure 1**. Distribution of UCA values of pregnant women who eventually gave birth

at term (n = 988), according to gestational age. Linear regression analysis showed a significant change in the range of UCA values from 16^+0^ to 23^+6^ weeks gestation (increase of 2.20 degrees per week, p <0.001).

Based on the normal distribution, Table 1 presents the mean values and corresponding values for the 5^rd^, 10^th^, 25^th^, 50^th^, 75^th^, 90^th^, 95^th^ percentile curves.

**Table 1**. UCA corresponding to 5^rd^, 10^th^, 25^th^, 50^th^, 75^th^, 90^th^, 95^th^ percentile according to GA (n=988).

| **GA** | **N** | **SD** | **Distribution of UCA according to percentile** | | | | | | |
| --- | --- | --- | --- | --- | --- | --- | --- | --- | --- |
|  |  |  | **5%** | **10%** | **25%** | **50%** | **75%** | **90%** | **95%** |
| 16^+0^ **-** 16^+6^ | 111 | 21.98 | 39.81 | 48.09 | 59.28 | 73.16 | 87.31 | 101.02 | 113.21 |
| 17^+0^ **-** 17^+6^ | 105 | 24.37 | 44.74 | 50.39 | 60.51 | 77.63 | 89.10 | 105.91 | 121.81 |
| 18^+0^ **-** 18^+6^ | 147 | 26.61 | 29.92 | 45.24 | 64.21 | 79.59 | 99.45 | 113.37 | 118.97 |
| 19^+0^ **-** 19^+6^ | 81 | 20.12 | 57.99 | 66.21 | 73.15 | 85.32 | 101.22 | 113.53 | 125.46 |
| 20^+0^ **-** 20^+6^ | 99 | 21.51 | 46.1 | 53.94 | 69.93 | 83.66 | 96.77 | 112.49 | 125.8 |
| 21^+0^ **-** 21^+6^ | 128 | 21.30 | 52.20 | 57.91 | 74.06 | 86.74 | 101.54 | 114.34 | 119.64 |
| 22^+0^ **-** 22^+6^ | 201 | 25.80 | 42.90 | 52.78 | 71.91 | 88.51 | 104.55 | 118.46 | 124.87 |
| 23^+0^ **-** 23^+6^ | 116 | 23.74 | 51.73 | 58.71 | 74.34 | 89.19 | 105.10 | 120.97 | 131.26 |

The range of the UCA at the 5^rd^ to 95^th^ percentile ranges from 45.15° (95% CI, 41.18° - 48.00°) to 123.00° (95% CI, 119.62° - 126.76°).
